# Does an endocrinology subspecialty residency rotation enhance resident endocrine clinical knowledge?

**DOI:** 10.1186/s12909-022-03110-6

**Published:** 2022-01-21

**Authors:** Yeng M. Miller-Chang, Jacqueline L. Gauer, Logan Butler, Andrew P.J. Olson, Rupendra T. Shrestha, J. Bruce Redmon

**Affiliations:** 1 Department of Health Sciences Technology, University of Minnesota Medical School, Minneapolis, USA; 2 Medical Education Outcomes Center, University of Minnesota Medical School, Minneapolis, USA; 3 Departments of Medicine and Pediatrics, University of Minnesota Medical School, Minneapolis, USA; 4 Department of Endocrinology, Park Nicollet Health Services, Park Nicollet Blvd, St Louis Park, Minnesota USA; 5 Division of Diabetes, Endocrinology and Metabolism, Department of Medicine, University of Minnesota Medical School, MMC 101 420 Delaware St SE, MN 55455 Minneapolis, USA

**Keywords:** Subspecialty rotation, Internal medicine, Endocrinology, In-service training exam

## Abstract

**Background:**

Internal Medicine (IM) programs offer elective subspecialty rotations in which residents may enroll to supplement the experience and knowledge obtained during general inpatient and outpatient rotations. Objective evidence that these rotations provide enhanced subspecialty specific knowledge is lacking. The purpose of this study was to determine whether exposure to an endocrinology subspecialty rotation enhanced a resident’s endocrinology-specific knowledge beyond that otherwise acquired during IM residency.

**Methods:**

Data were collected on internal medicine resident scores on the American College of Physicians Internal Medicine In-Training Examinations (IM-ITE) for calendar years 2012 through 2018 along with enrollment data as to whether residents had completed an endocrinology subspecialty rotation prior to sitting for a given IM-ITE. Three hundred and six internal medicine residents in the University of Minnesota Internal Medicine residency program with 664 scores total on the IM-ITE for calendar years 2012 through 2018. Percentage of correct answers on the overall and endocrine subspecialty content areas on the IM-ITE for each exam were determined and the association between prior exposure to an endocrinology subspecialty rotation and percentage of correct answers in the endocrinology content area was analyzed using generalized linear mixed-effects models.

**Results:**

Two hundred and thirty-three residents (76%) completed an endocrinology subspecialty rotation at some point during their residency; 121 (40%) residents had at least one IM-ITE both before and after exposure to an endocrine subspecialty rotation. Exposure to an endocrinology subspecialty rotation exhibited a positive association with the expected IM-ITE percent correct on the endocrinology content area (5.5% predicted absolute increase). Advancing year of residency was associated with a predicted increase in overall IM-ITE score but did not improve the predictive model for endocrine subspecialty score.

**Conclusions:**

Completion of an endocrinology subspecialty elective was associated with an increase in resident endocrine specific knowledge as assessed by the IM-ITE. These findings support the value of subspecialty rotations in enhancing a resident’s subspecialty specific medical knowledge.

## Background

In the United States, following four years of medical school newly graduated physicians who wish to practice in the specialty of Internal Medicine (IM) (or one of its subspecialties) first complete an additional three post-graduate years of IM specific training in an accredited IM residency program. In the US, these programs are accredited by the Accreditation Council for Graduate Medical Education (ACGME) which sets standards for residency and fellowship programs and the institutions that sponsor them. As part of the ACGME standards, residency programs are charged with the task of preparing residents with the knowledge and skills to practice independently. Such programs must also provide regular objective assessment of resident competence [1]. Residents must demonstrate knowledge of the core content of IM subspecialties seen in a general medical practice for successful entry into subspecialty fellowship training programs, including subspecialties which are primarily outpatient and office-based. Training in outpatient subspecialties can occur during rotations in general outpatient clinics, including resident continuity clinics. In the inpatient setting, resident clinical experience and education in primarily outpatient subspecialties may be limited.

To alleviate such limitations, IM programs offer elective rotations in specific subspecialty areas, allowing for focused clinical experience and education in a particular subspecialty. However, whether these elective rotations provide enhanced knowledge in the subspecialty beyond knowledge acquired during typical inpatient rotations or outpatient general medicine clinics is either unknown or is based on residents’ own perceptions of their knowledge [2].

The annual American College of Physicians (ACP) Internal Medicine In-Training Examination (IM-ITE) provides a standardized assessment of resident knowledge in eleven content areas including general medicine [3]. First offered in 1988, the exam is elective and residency programs can decide on an individual basis whether to offer it to their residents or not. However currently approximately 98% of US IM residents take the exam each year [4]. The IM-ITE consists of 300 questions; approximately 15% of these are in the general internal medicine content area and approximately 6% are in the endocrinology content area. Although questions are directed at residents in post graduate year two of residency (PGY-2), most programs, including the University of Minnesota program, opt to have all residents take the exam during each year of residency. The exam is developed each year by a question-writing committee composed of experts in general internal medicine or one of the medical subspecialties. Questions follow a patient-based clinical scenario format similar to the American Board of Internal Medicine Certifying Examination (ABIM-CE) and residents complete the exam in one sitting [4].

Attesting to the validity of the exam, resident scores have been shown to increase with increasing time in training and overall scores on the IM-ITE predict subsequent performance on the ABIM-CE [5]. Test reliability is high (KR_20_ >0.90) across administrations similar to national certifying exams [6]. In-training examination (ITE) scores have been reported to be better predictors of subsequent resident scores on subspecialty certification exams than ratings of medical knowledge from program directors [7]. Since the IM-ITE is a national, standardized exam residents take every year, the IM-ITE can serve as a measure of both knowledge of general IM and IM subspecialties.

Targeted curricula have previously been reported to improve overall IM-ITE scores. Mathis et al. compared overall IM-ITE scores between PGY-2 and PGY-3 residents before and after implementation of a structured 12 month program of multiple choice tests and board review type questions. IM residents exposed to the program showed a significantly greater improvement in PGY-2 to PGY-3 ITE percentile scores compared to the unexposed historical control group [8]. Similarly, Sisson et al. found that IM residents who completed a greater number of didactic modules of the Johns Hopkins Internal Medicine Curriculum scored higher on the IM-ITE [9].

With regard to the effect of more targeted, topic specific curriculum interventions, Trickey et al. reported that an intervention of research and statistics lectures provided to surgery residents resulted in a significant improvement in scores for the research and statistics items on the American Board of Surgery ITE [10]. Implementation of an evidence based medicine curriculum for pediatric emergency medicine fellows was shown to improve subsequent scores on the scholarly activities subsection of the American Board of Pediatrics ITE [11].

To our knowledge, the effectiveness of IM subspecialty rotations to improve corresponding IM-ITE content area scores has not been studied. For the current study, we sought to determine whether IM residents who completed an endocrinology subspecialty elective rotation subsequently scored a higher percentage correct (“score”) on the endocrinology content area of the IM-ITE in comparison to their peers who had not enrolled in the rotation prior to the exam.

## Methods

### Institutional approval

The study was approved as exempt from review by the University of Minnesota Institutional Review Board (IRB ID: STUDY00004872).

### Participants and measures

The University of Minnesota IM residency program offers a four-week endocrinology elective rotation residents can take at any point during their three-year residency. Residents can choose to do the rotation at one of three teaching hospitals and their affiliated outpatient specialty clinics: (1) the University of Minnesota Medical Center, the primary academic teaching hospital; (2) the Minneapolis VA Medical Center; and (3) the HealthPartners Regions Hospital in St. Paul, MN.

The educational curriculum at all sites includes clinical experience in both the outpatient and inpatient settings. At the University of Minnesota site, residents receive 8 h of scheduled didactic lectures delivered by faculty in the areas of type 1 and type 2 diabetes; hyperlipidemia; osteoporosis and calcium disorders; and disorders of pituitary, gonadal, adrenal, and thyroid function. At the Minneapolis VA Medical Center, residents receive didactic lectures in the areas of thyroid and adrenal disorders, osteoporosis, and obesity. At the Regions Hospital site, the educational experience is primarily delivered through informal teaching by faculty in the outpatient clinics and inpatient consult settings.

All IM residents take the IM-ITE during each year of their residency. For each academic year from 2012 to 2018, we collected IM-ITE overall and endocrinology content area scores (reported as percent correct) for each resident and determined whether the resident completed an endocrinology subspecialty elective rotation over the course of residency prior to taking the exam for the given year.

The University of Minnesota’s Medical Education Outcomes Center (MEOC) merged resident IM-ITE score data with resident demographic data from the University of Minnesota’s Department of Medicine resident management systems.

Overall, 306 residents (Table 1) with a total of 664 IM-ITE scores comprise the study cohort. Of the 306 residents, 233 (76%) residents completed an endocrinology subspecialty rotation at some point during their residency, and 121 (40%) residents had taken at least one IM-ITE both before and after completing an endocrinology rotation. Of the 664 IM-ITE scores, 222 (33%) resulted after residents completed an endocrinology rotation. Table 2 shows overall and endocrinology content area IM-ITE scores by rotation status and post-graduate year (PGY) level.

**Table 1:** Count and percentage of University of Minnesota Internal Medicine residents from academic years 2012 through 2018 by number of IM-ITE exams (*n* = 306)

Number of exams per resident	Count of residents (%)
One exam	86 (28.1%)
Two exams	86 (28.1%)
Three exams	133 (43.5%)
Four exams	1 (0.3%)^a^

Abbreviations: *IM-ITE* Internal Medicine In-Training Examination; *PGY* Post-Graduate Year

^a^One resident spent two years in PGY-2, thus had taken the IM-ITE twice for PGY-2

**Table 2:** Statistics on University of Minnesota Internal Medicine residents’ 664 IM-ITE exams from academic years 2012 through 2018 by PGY level and endocrinology rotation enrollment status prior to IM-ITE sitting

	PGY-1			PGY-2			PGY-3		
Endocrinology rotation prior to IM-ITE sitting	Yes	No	All Residents	Yes	No	All Residents	Yes	No	All Residents
Count (% of PGY level)	11 (4.7%)	222 (95.3%)	233	72 (31.7%)	155 (68.3%)	227	139 (68.1%)	65 (31.9%)	204
Overall IM-ITE mean score (SD)^a^	57.1% (6.1%)	62.4% (8.2%)	62.2% (8.2%)	69.6% (7.7%)	69.4% (8.1%)	69.5% (8.0%)	73.5% (7.6%)	69.6% (7.6%)	72.3% (7.8%)
IM-ITE endocrinology content area mean score (SD)^a^	68.1% (10.3%)	64.7% (13.2%)	64.9% (13.1%)	71.2% (12.6%)	68.4% (14.0%)	69.3% (13.6%)	71.4% (13.2%)	65.7% (11.7%)	69.6% (13.0%)

Abbreviations: *SD* standard deviation; *IM-ITE* Internal Medicine In-Training Examination; *PGY* Post-Graduate Year

^a^The score is defined as the percentage of correct answers

### Statistical analysis

Conventional hypothesis tests on linear regression coefficients, such as the t-test and ANOVA, assume independence of all response variable measurements [12]. Assuming independence of the response variable (i.e., the overall IM-ITE score or the endocrinology content area IM-ITE score) is inappropriate for the hypotheses of interest, given that IM-ITE exams in a given year are identical (“year dependence”). In addition, residents take the IM-ITE multiple times, making each resident’s overall and endocrinology content area IM-ITE scores inherently dependent (“student dependence”).

To incorporate these sources of dependence, we used generalized linear mixed-effects models (GLMM) with normal random component and logit link to analyze the association of a resident’s years of residency and exposure to an endocrinology elective rotation with either a resident’s overall expected IM-ITE score or the resident’s expected endocrinology content area IM-ITE score [13]. For each GLMM considered, either the overall IM-ITE score or the endocrinology content area IM-ITE score (expressed as a number between 0 and 1) was the response variable. To avoid division-by-zero errors with the logit link, scores were truncated from above at 0.999. Truncation was only applied for the purposes of modeling and was not applied in Table 2.

We considered the following fixed-effect covariates for each GLMM: (1) a fixed effect denoting whether the student took the IM-ITE exam after an endocrinology rotation (coded as 1 if so, 0 if not), (2) a fixed effect denoting the PGY level at the time of the IM-ITE (coded as 1, 2, 3 for residency levels 1 through 3 respectively), and (3) a fixed effect for the interaction of these two variables. In each model, we included two random effects: one incorporating year dependence of IM-ITE exams, and another to incorporate student dependence. Data for PGY-4 and PGY-5 were excluded due to small sample sizes.

We used *P* < 0.01 to denote statistical significance of model parameters. We used R 4.0.2 (The R Foundation; Vienna, Austria) to perform all computations.

## Results

With regard to overall IM-ITE scores, we found the GLMM using PGY level as the only fixed effect provided the best fit to the overall IM-ITE score. Table 3 contains the parameters for this model. With this model, a one-year increase in PGY level has an association with a 5.4–5.9% absolute increase in the overall IM-ITE score.

Table 3 also shows the parameters for the model which provided the best fit to the IM-ITE endocrinology content area score. In contrast to the model for overall ITE scores, the model using only endocrinology rotation status provided the best fit of the endocrinology content area IM-ITE score and does not include PGY level. Applying this model, taking an IM-ITE after the endocrinology rotation has an association with a 5.5% absolute increase in the endocrinology content area IM-ITE score.

**Table 3:** Generalized linear-mixed effects model parameters by response variable

Overall IM-ITE score				
Fixed effect	Estimate	SE	t	P
Intercept	0.274	0.054	5.01	< 0.01
PGY level	0.266	0.008	33.04	< 0.01
IM-ITE endocrinology content area score				
Fixed effect	Estimate	SE	t	P
Intercept	0.832	0.145	5.73	< 0.01
IM-ITE after endocrinology rotation	0.276	0.046	6.01	< 0.01

Abbreviations: *IM-ITE* Internal Medicine In-Training Examination; *PGY* Post-Graduate Year

## Discussion

We found a positive association between exposure to an endocrinology subspecialty rotation and expected subsequent performance on the endocrinology content area of the IM-ITE. We found no evidence of an association between exposure to the endocrinology subspecialty rotation and expected performance on the overall IM-ITE, suggesting that the rotation enhanced endocrinology-specific knowledge. Conversely, adding PGY level did not result in an improved model for the expected endocrinology content area score. Thus, completion of an endocrinology elective rotation appears to enhance resident knowledge in endocrinology beyond that otherwise attained during other resident experiences.

As expected, there is evidence of a positive association between PGY level and the overall IM-ITE score. In our cohort, each year of residency has an association with an absolute increase in the expected overall IM-ITE score of 5.4–5.9%. These values are consistent with previous studies which have reported increases in resident ITE scores with each progressing year of residency [6, 14]. Interestingly, in our data, completing an endocrine subspecialty rotation appears to have contributed a similar amount of improvement to endocrine subscale performance as completing one year of residency training contributed to overall ITE performance.

We are not aware of prior studies that examine the effect of subspecialty rotations or other factors which impact performance on IM-ITE content area scores. Prior studies have demonstrated evidence of an association between overall IM-ITE performance and age at start of residency, US versus international medical-school training, conference attendance, and self-directed use of online resources [15]. In addition, specific interventions such as a rotation-specific multiple-choice test/board review program and use of an online ambulatory medicine curriculum have been associated with improvement in overall IM-ITE scores [8, 9]. However, these studies did not address the effects of interventions on subspecialty specific medical knowledge. As noted earlier, studies in the surgery and pediatric emergency medicine specialties have reported the effect of interventions on more topic specific areas; however, these interventions focused on research and statistics and evidence based medicine rather than medical knowledge per se.

Our study does not allow us to identify what individual elements of an endocrine subspecialty rotation result in improved scores on the endocrine subspecialty section of the ITE. Potential elements include exposure to structured endocrine didactic lectures provided by endocrine faculty, increased exposure to endocrine disorders through participation in endocrine outpatient clinics and inpatient consults, exposure to weekly endocrine teaching, research and clinical conferences during the rotation and possibly resident initiative to increase outside reading on endocrine topics during the rotation. It is possible that all of these elements play a role.

A limitation of our study is that residents were not randomized to enroll or not enroll in the endocrinology subspecialty rotation during their residency; enrollment in the rotation was voluntary. Therefore, it is possible that residents electing to take an endocrinology rotation were residents more interested in endocrinology and were more motivated than their counterparts to perform well on the endocrinology IM-ITE content area. However, 233 (76%) residents did have the rotation at some point during residency, and 121 (40%) residents had IM-ITE scores both before and after an endocrinology rotation, allowing us to make resident-level comparisons before and after residents enrolled in a rotation. In addition, we did not see evidence of a difference in overall IM-ITE scores at any given PGY level between exposed and non-exposed residents.

We see the purpose of subspecialty rotations during IM residency training as threefold: (1) to expose residents to the subspecialty field as a potential career path, (2) to provide clinical experience in the subspecialty at a level they might not encounter during inpatient hospital rotations or in the primary care clinic setting, and (3) to provide a basic level of knowledge in the field that should be attained by all internists. Our results suggest that subspecialty rotations can help to achieve this last purpose.

## Conclusions

We found in our program that exposure to an endocrinology subspecialty rotation was associated with an improvement in endocrinology-specific knowledge as assessed by resident performance on the IM-ITE. Our findings support the value of subspecialty rotations as part of an IM resident’s postgraduate education. IM residency programs may want to use similar methods and metrics as one way to document the effect of their curriculum on resident medical knowledge competency.

## Data Availability

The datasets analyzed during the current study are available from the corresponding author on reasonable request.

## Abbreviations

IM: Internal Medicine
IM-ITE: Internal medicine in-training examination
ACGME: Accreditation Council for Graduate Medical Education
ACP: American College of Physicians
PGY: Post-graduate year
ABIM-CE: American Board of Internal Medicine certifying examination
GLMM: Generalized linear mixed-effects model
